# Estimated Cost of Circulating Tumor DNA for Posttreatment Surveillance of Human Papillomavirus–Associated Oropharyngeal Cancer

**DOI:** 10.1001/jamanetworkopen.2021.44783

**Published:** 2022-01-25

**Authors:** Roman O. Kowalchuk, Benjamin C. Kamdem Talom, Kathryn M. Van Abel, Daniel M. Ma, Mark R. Waddle, David M. Routman

**Affiliations:** 1 Department of Radiation Oncology, Mayo Clinic, Rochester, Minnesota; 2 Department of Otolaryngology, Mayo Clinic, Rochester, Minnesota

## Abstract

This cross-sectional study compares the cost and effectiveness of 3 posttreatment strategies for surveillance of human papillomavirus (HPV)–associated oropharyngeal cancer.

## Introduction

Because of the favorable treatment outcomes for human papillomavirus (HPV)–associated oropharyngeal squamous cell cancer (OPSCC), efforts are ongoing to reduce the burden and cost of posttreatment surveillance. National Comprehensive Cancer Network (NCCN) guidelines recommend 1 imaging study (positron emission tomography/computed tomography [PET/CT] preferred) 8 to 12 weeks after completion of radiotherapy and/or chemotherapy, but additional imaging studies are commonly ordered.^1^ Circulating tumor DNA (ctDNA) has shown impressive predictive value in surveillance across multiple malignant neoplasms, with positive and negative predictive values of 94% and 100%, respectively, for HPV-associated OPSCC.^2^ We undertook a cross-sectional cost comparison of ctDNA utilization for posttreatment surveillance of HPV-associated OPSCC, which may offer equivalent or even improved accuracy compared with existing imaging strategies.

## Methods

First, we obtained the costs of various aspects of surveillance testing at the institutions the *US News & World Report* designated as “top 10 cancer centers” for 2021-2022 (Table). Procedure prices were obtained from each center’s website, and the median prices for 2021 were used for the analysis. Cost of ctDNA was estimated at $500 per test, consistent with similar tests, such as the Epstein-Barr virus DNA test. This analysis was limited to patients with detectable pretreatment blood samples (approximately 90% of patients). This study was deemed exempt from institutional review board (IRB) approval by the Mayo Clinic IRB because the analysis used no patient data. The Mayo Clinic IRB also waived informed patient consent for the same reason. All components of the analysis used publicly available data. Our study followed the Strengthening the Reporting of Observational Studies in Epidemiology (STROBE) reporting guideline for cross-sectional studies.

**Table.** Median Costs for Various Aspects of Posttreatment Surveillance

Aspect of posttreatment surveillance	Median cost (IQR), $
PET/CT	8240 (7290-8710)
Nasopharyngoscopy^a^	303
Biopsy	5990 (2610-7600)
CT of head and neck with contrast	2510 (2260-3030)
Follow-up visit	265 (256-525)
Surveillance scenario^b^	
Scenario 1: NCCN-recommended	12 780 (11 765-15 331)
Scenario 2: imaging-intensive	29 258 (26 347-32 754)
Scenario 3: ctDNA	8541 (8474-10 620)

Abbreviations: CT, computed tomography; ctDNA, circulating tumor DNA; NCCN, National Comprehensive Cancer Network; PET/CT, positron emission tomography/CT.

^a^ The cost of nasopharyngoscopy is based on the Medicare reimbursement rate, with a 2.64 multiplier. This multiplier was used because the contacted cancer centers provided the cost of nasopharyngoscopy only as part of a bundle payment including other costs.

^b^ Scenario 1 includes a PET/CT at 3 months after radiotherapy followed by a follow-up visit and nasopharyngoscopy every 3 months for 2 years. Scenario 2 adds another PET/CT at 1 year and at 2 years. Scenario 3 replaces surveillance imaging with ctDNA testing every 3 months.

From May 1 to October 8, 2021, 3 surveillance scenarios were analyzed and the corresponding costs computed for 2 years. Scenario 1 included the NCCN-recommended PET/CT at 3 months after radiotherapy, followed by a follow-up visit and nasopharyngoscopy every 3 months for 2 years. The imaging-intensive scenario 2 incorporated another PET/CT at 1 year and at 2 years, as well as all testing from scenario 1. Scenario 3 replaced surveillance imaging with ctDNA testing every 3 months but maintained follow-up and nasopharyngoscopy every 3 months for 2 years. Equivocal results prompted repeat imaging or ctDNA.^2^ Concerning findings led to follow-up and imaging (CT with contrast for PET/CT surveillance and PET/CT for ctDNA surveillance). Concerning findings for ctDNA were defined as 2 positive tests; therefore, the cost of a second ctDNA test was also included. The analyses were conducted using Excel 2013 (Microsoft Corp).

## Results

The top 10 cancer centers in the United States in 2021, according to *US News & World Report*, were considered for the cost data. Use of ctDNA was the lowest-cost strategy ($8541); even if the cost of ctDNA were doubled, it would remain the least costly strategy. Results also supported the low cost of ctDNA in cases of potential recurrence. The most striking difference arose in the equivocal scenario, which could simply be addressed with a repeated ctDNA test for ctDNA-based surveillance, for a final cost of $9041 (Figure). Conversely, equivocal and concerning intensive-imaging findings each led to similar cost increases on the order of $8000, from a base cost of $29 258 to $37 497 for equivocal results and $38 026 for concerning results. However, these totals may be underestimates when follow-up visits are added.

**Figure.**
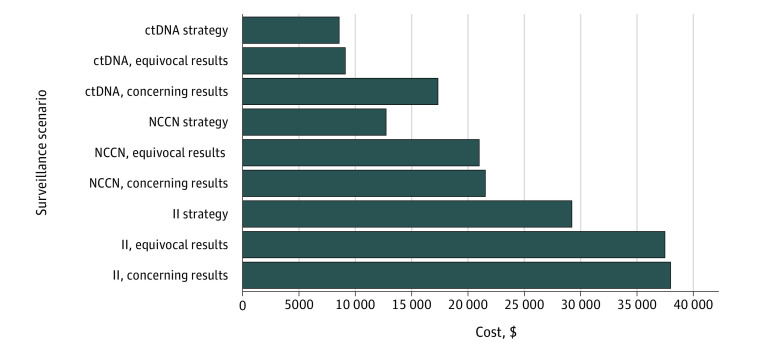
Cost Comparison of False-Positive Findings in 3 Surveillance Imaging Scenarios The 3 scenarios were circulating tumor DNA (ctDNA), National Comprehensive Cancer Network (NCCN) guideline, and imaging-intensive (II) strategies. The scenarios also considered when equivocal results resulted in repeated imaging (II, equivocal) or ctDNA (ctDNA, equivocal) for the imaging-based and ctDNA-based strategies, respectively. Concerning findings prompted follow-up and imaging (computed tomography [CT] with contrast for positron emission tomography [PET]/CT surveillance [II, concerning] and PET/CT for ctDNA surveillance [ctDNA, concerning]). Concerning findings for ctDNA were defined as 2 positive test results; thus, the cost of a second ctDNA test was also included.

## Discussion

Overall, ctDNA may offer accuracy that is comparable to conventional surveillance imaging for HPV-associated OPSCC, but with substantially lower associated costs.^2^ Its use could markedly reduce the cost of false-positive PET/CT results, with a positive predictive value of approximately 50%.^3^ Overall, 5% to 10% of patients may have a false-positive PET/CT result.^4^ These values are particularly striking since only 10% to 15% of patients with HPV-associated OPSCC will develop recurrent disease.^5^ In addition, fewer imaging studies would reduce costs and ameliorate patient anxiety.^6^ Because ctDNA is a laboratory test, surveillance could be completed without a follow-up visit, further reducing costs and travel burden. Also, ctDNA offers a potential lead time of more than 3 months prior to biopsy-proven recurrence.^2^ We strongly encourage further biomarker studies to involve ctDNA for posttreatment surveillance. Validation in HPV-associated OPSCC is underway (NCT04564989). Limitations of this analysis include the lack of a comparison of incremental cost-effectiveness ratio, which is a result of the limited literature available in this field. Our analysis supports ctDNA as a low-cost posttreatment surveillance strategy for HPV-associated oropharyngeal cancer compared with imaging-based strategies.
